# *Porphyromonas gingivalis* disturbs host–commensal homeostasis by changing complement function

**DOI:** 10.1080/20002297.2017.1340085

**Published:** 2017-06-30

**Authors:** Ingar Olsen, John D. Lambris, George Hajishengallis

**Affiliations:** ^a^ Department of Oral Biology, Faculty of Dentistry, University of Oslo, Oslo, Norway; ^b^ Department of Pathology and Laboratory Medicine, Perelman School of Medicine, University of Pennsylvania, PA, USA; ^c^ Department of Microbiology, School of Dental Medicine; University of Pennsylvania, PA, USA

**Keywords:** *P. gingivalis*, keystone pathogen, commensal microbiota, complement, animal model, periodontitis

## Abstract

*Porphyromonas gingivalis* is a Gram-negative anaerobic rod that has been proposed as an orchestrator of complement-dependent dysbiotic inflammation. This notion was suggested from its capacities to manipulate the complement–Toll-like receptor crosstalk in ways that promote dysbiosis and periodontal disease in animal models. Specifically, while at low colonization levels, *P. gingivalis* interferes with innate immunity and leads to changes in the counts and composition of the oral commensal microbiota. The resulting dysbiotic microbial community causes disruption of host–microbial homeostasis, leading to inflammatory bone loss. These findings suggested that *P. gingivalis* can be considered as a keystone pathogen. The concept of keystone pathogens is one where their effects have community-wide significance and are disproportionate of their abundance. The present review summarizes the relevant literature and discusses whether the results from the animal models can be extrapolated to man.

## Introduction

**Figure 1.**
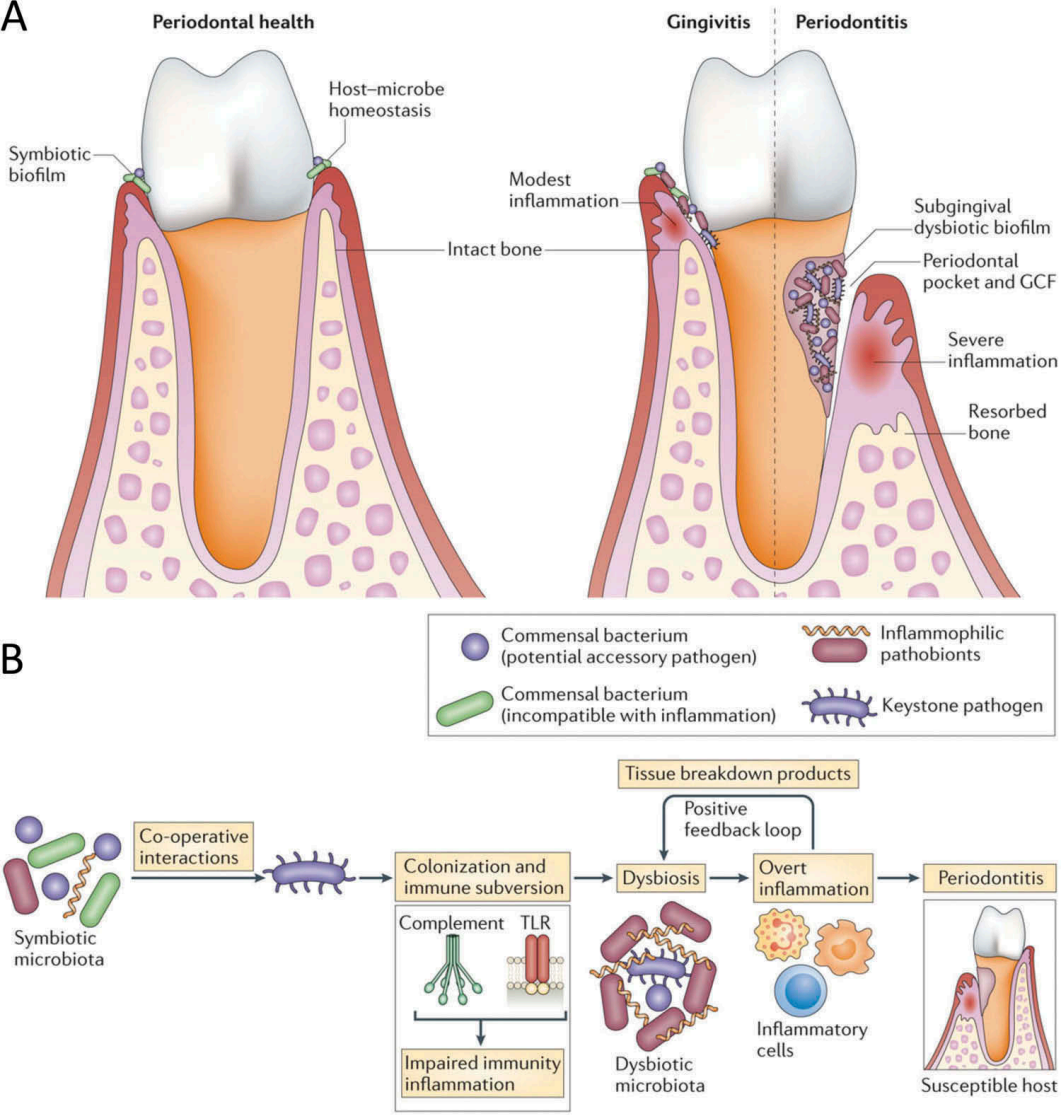
Dysbiosis and periodontal disease. (A) Progression from a state of periodontal health and host–microbe homeostasis to gingivitis (periodontal inflammation without bone loss) and to periodontitis, associated with a dysbiotic biofilm, formation of periodontal pockets, and induction inflammatory bone loss. (B) Periodontitis is induced in susceptible hosts by a polymicrobial community, wherein different members fulfill distinct roles that converge synergistically to cause destructive inflammation. Keystone pathogens, the colonization of which is facilitated by accessory pathogens, initially subvert the host response, leading to a dysbiotic community where pathobionts over-activate the inflammatory response and induce periodontal tissue degradation, including resorption of the supporting alveolar bone. Inflammation and dysbiosis positively reinforce each other because inflammatory tissue breakdown products (e.g. collagen peptides, heme-containing compounds) carried in the pockets *via* the gingival crevicular fluid are used as nutrients by the dysbiotic microbiota. This process generates a self-perpetuating pathogenic cycle that may underlie the chronicity of periodontitis. (From Hajishengallis [23]. Used with permission.)

Bacteria that colonize subgingival sites of the teeth are implicated in periodontal disease (Figure 1A), although their precise roles and mechanisms have been a matter of debate, reflecting different theories over the years [1–3]. Recent human microbiome analyses and mechanistic studies in relevant preclinical models suggest that periodontal disease is a dysbiotic disease rather than a bacterial infection [4,5]. In the classical sense of the term, infections are initiated by specific exogenous pathogens and follow Koch’s postulates. In contrast, periodontitis is not caused by a single or even a select few bacterial species, historically designated ‘periopathogens’. Indeed, the microbial etiology of periodontitis entails synergistic interactions between different indigenous species with distinct roles in the microbial community, leading to dysbiosis (Figure 1B). Dysbiosis involves changes in the abundance or influence of individual species within a polymicrobial community (relative to their abundance or influence in health), leading to altered host–microbial interactions and destructive inflammation. Bacteria termed ‘keystone pathogens’ manipulate the host response and undermine immunity and host–microbe homeostasis, thereby leading to dysbiosis [6]. Certain commensals, though non-pathogenic by themselves in the oral environment, can promote keystone pathogen metabolic activity and colonization and, as such, are implicated in periodontitis as ‘accessory pathogens’. When homeostatic mechanisms fail, bacteria known as ‘inflammophilic pathobionts’ further exacerbate inflammation, creating a nutritionally conducive environment where they can flourish (Figure 1B). Specifically, inflammatory breakdown products of connective tissue are released into the gingival crevicular fluid that bathes the periodontal pockets and are utilized as nutrients by certain bacterial species (e.g. proteolytic and asaccharolytic), which can therefore expand at the expense of other species that cannot capitalize on the new environmental conditions [4,7]. Importantly, destructive inflammation and dysbiosis engage in a self-sustained feed-forward loop. Indeed, since products of inflammatory tissue destruction (e.g. degraded collagen peptides and heme-containing compounds) are used as nutrients by certain species in the community, these can exhibit further growth and persist, thereby exacerbating inflammation and contributing to the chronicity of periodontitis.

These new concepts have been integrated in a newly proposed model for periodontal disease pathogenesis known as ‘polymicrobial synergy and dysbiosis’ (PSD). According to the PSD model, the periodontal host response is initially subverted by keystone pathogens, the colonization and metabolic activities of which are assisted by accessory pathogens, and is subsequently over-activated by pathobionts, leading to destructive inflammation in susceptible hosts (Figure 1B) [5,8]. Susceptibility to periodontitis, hence the transition from host–microbe symbiosis to dysbiosis and disease, is determined by a variety of factors (genetic; epigenetic; environmental, such as smoking, stress, and diet; systemic diseases, such as diabetes; and aging) that may modify the host response in either a protective or a destructive direction.

From the above, one may derive that the commensal or pathogenic properties of bacteria are not intrinsic but rather contextual features. In other words, such properties should be considered within the context of both the microbial community and the host immune status. The health- or disease-associated properties of an organism essentially represent a spectrum from commensalism to pathogenicity (including newly recognized categories such as those discussed above) and cannot be described in simple dichotomous terms (i.e. commensals vs. pathogens). Overall, the collective pathogenic potential of a microbial community, termed nososymbiocity, depends upon host susceptibility and the outcome of interbacterial interactions [9].

**Figure 2.**
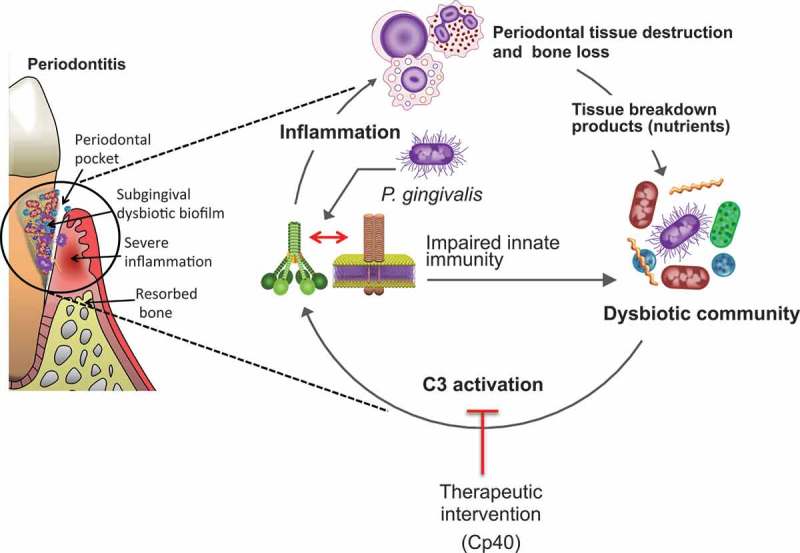
Complement involvement in *Porphyromonas gingivalis*–induced dysbiosis and inflammation. At low colonization levels, *P. gingivalis* can act as a keystone pathogen that manipulates complement–Toll-like receptor crosstalk, leading to the dysbiotic transformation of the microbiota (increased counts and altered composition). In dysbiosis, pathobionts over-activate the inflammatory response in a complement C3-dependent manner, resulting in destructive periodontal inflammation and bone loss. Inflammation and dysbiosis create a feed forward loop, which is essentially a disease-provoking vicious cycle. Therapeutic intervention at the C3 level with a specific inhibitor (Cp40) appears to break the cycle and inhibits periodontitis in non-human primates. (From Mastellos et al. [92]. Used with permission.)

Much of what is known regarding the basic mechanistic aspects of periodontal dysbiosis is derived from studies of *Porphyromonas gingivalis*–induced periodontitis in mouse models of periodontitis. Such studies have shown that *P. gingivalis* acts as a keystone pathogen that exploits complement function to orchestrate dysbiosis and precipitate periodontitis (Figure 2). In this review, the relevant literature is summarized and discussed, and comment is made on whether the results from the mouse model can be extrapolated to human periodontitis. It would be instructive to start with a short background on the complement system.

## The complement system

The complement system is a major part of the innate immune system and is responsible for the host defense against invading microorganisms, inflammation, and homeostasis [10,11]. Being composed of >50 interacting serum circulating and cell-surface receptors and regulators, complement provides an effective and early immune surveillance. Indeed, complement is the first defense line in the host against invading microbes. These are sensed by soluble pattern-recognition molecules, such as pentraxins, ficolins, collectins (e.g. the mannose-binding lectin of the complement system), and the complement component C1q [12], as well as innate pattern-recognition receptors such as Toll-like receptors (TLRs), which cooperate with complement pathways [13]. The complement system has important roles in labeling bacteria for phagocytosis, killing them by pore formation and stimulating B cells *via* C3d. Complement also has a heavy impact on T cells, and thus it has been considered a major bridge between innate and adaptive immunity [13,14]. To resist infection, optimal crosstalk between the complement system and TLRs is needed [13]. Complement factors are predominantly produced in the liver but also locally in tissues. The role of complement in controlling immune responses against invading pathogens (‘outside’ function) has long been appreciated. Also, new and unexpected functions of complement have been discovered, for example driving of the cellular machinery for initiation and regulation of T-effector cell responses (‘inside’ function) [14].

Three pathways are involved in activation of the complement system (classical, lectin, and alternative). The results of activation are opsonization of microorganisms for phagocytosis, production of chemoattractants, and lysis of targeted susceptible Gram-negative bacteria [15]. In brief, activation of the classical pathway occurs when IgG or IgM antibody attached to the bacterial surface is recognized by binding of the C1 complex (Clq, Clr, and C1s). Clr activates C1s, which cleaves C4 and C2, thereby producing the classical pathway C3 convertase. The C3 convertase cleaves C3, producing C5 convertase and release of C3a. The C5 convertase cleaves C5 into C5a and C5b. C3a and C5a are anaphylatoxins and have powerful effects in mediating inflammation, modulating adaptive immunity, and repairing regenerative processes [10]. Activation of the classical pathway can also take place independently of antibodies, and C1q can bind directly to certain microbial molecules [10].

The lectin pathway is quite similar to the classical pathway except for its initial steps. It is initiated by binding of the complex of mannose-binding lectin and the serine proteases mannose-binding lectin associated proteases −1 and −2 (MASP-1/2) to mannose groups on the surface of invading pathogens [10]. MASP-1 activates MASP-2, which acts like C1s in the classical pathway, producing C3 convertase. The remaining steps follow the classical pathway.

The alternative pathway is initiated by spontaneous hydrolysis of C3, deposition of C3b on the surface of activating surfaces, and release of C3a with generation of various biological effector molecules. As long as the activation process is not inhibited by specialized regulatory proteins (physiologically present on host cells but not normally on microbial cell surfaces or other foreign surfaces), this initiation is followed by rapid propagation of the alternative pathway through an amplification loop. In the alternative pathway, factor B binds to C3b and is cleaved by factor D, producing the alternative pathway C3 convertase. Properdin binds to the convertase to stabilize it. C3 convertase cleaves C3, forming more C3a and the alternative pathway C5 convertase. The latter cleaves C5 to C5a and C5b [10,11].

The three pathways of the complement system converge in a terminal pathway. Here, C5b binds to C6, and C7 binds to the C5b–C6 complex. The newly formed C5b–C7 complex inserts into the target membrane after which C8 binds to the C5b–C7 complex and produces a small pore in the membrane. The membrane attack complex is formed by binding of C9 molecules to the C5b–C8 complex [10,11].

### P. gingivalis *as a keystone pathogen: evidence from mouse models*

Until recently, much research has been directed toward understanding virulence determinants and mechanisms of periodontal pathogens, such as the ‘red complex’ bacteria [16,17], in the context of a conventional host–pathogen interaction, as exemplified by diseases with defined infective etiology. However, recent studies in animal models, which are described below, suggested that periodontitis is not caused by individual pathogens but rather by a synergistic microbial community. In such a community, the role of *P. gingivalis* is to tip the balance from homeostasis to dysbiosis (Figure 2). Periodontitis may therefore fundamentally represent disruption of host–microbial homeostasis, where even commensal bacteria could opportunistically mediate destructive inflammation. This notion is consistent with the emerging association of previously underappreciated species (e.g. certain Gram-positive bacteria) with diseased sites in human periodontitis [18–22].

*P. gingivalis* is a Gram-negative anaerobic and asaccharolytic bacterium that has long been implicated in human periodontitis and is also suspected to play a role in the systemic diseases of man [23]. When *P. gingivalis* was inoculated orally into a specific-pathogen-free (SPF) validated mouse model of periodontitis, significant alveolar bone loss occurred after 6 weeks [24]. In contrast, similar inoculation of *P. gingivalis* into germ-free (GF) mice did not cause bone loss, even though GF and SPF mice were colonized by *P. gingivalis* to the same extent. This finding suggests that commensals were necessary for periodontal bone loss to occur. Interestingly, inoculation of the SPF mice with *P. gingivalis* was followed by increased levels of cultivable commensal oral bacteria and a change in the qualitative composition of the oral microbiota. These dysbiotic changes caused pathological bone loss in the *P. gingivalis*–colonized SPF mice. Intriguingly, the numbers of *P. gingivalis* constituted <0.01% of the total microbiota, as assessed with real-time polymerase chain reaction. Thus, although *P. gingivalis* constituted only a minor portion of the microbiota, it significantly altered the numbers and composition of the commensal bacteria, leading to dysbiosis [3]. However, *P. gingivalis* lost the ability to cause dysbiosis and pathological bone loss in complement C5a receptor-1 (C5aR1)-deficient mice, suggesting that complement is required in the disease process [24].

It should be noted that the ability of *P. gingivalis* to induce bone loss in the same model is enhanced when it is inoculated together with *Streptococcus gordonii* [25]. This can be attributed to the capacity of *S. gordonii* to provide metabolic and colonization support to *P. gingivalis*. In this regard, *P. gingivalis* and *S. gordonii* were found to up- or downregulate several metabolic pathways upon contact with each other, meaning that they were actively responding to each other in a community life-style [26,27]. Although *S. gordonii* has been viewed as a commensal in the oral cavity, it would be more accurately categorized as an accessory pathogen [28]. In a similar context, *Fusobacterim nucleatum* also provides metabolic support to *P. gingivalis* and has a positive impact on its biomass [4].

In ecology, the role of keystone species becomes particularly evident when they are removed from their communities, as the removal causes dramatic changes to the rest of the community [29]. Consistently, when *P. gingivalis* was selectively removed from the oral cavity of mice by using a C5aR1 antagonist, the dysbiosis was reversed and inflammation ceased [24]. The fact that a C5aR1 antagonist eradicated *P. gingivalis* from periodontal tissue and inhibited disease progression suggested that elimination of a keystone pathogen can, at least in principle, be used in the treatment of periodontal disease.

In the absence of *P. gingivalis*, the commensal microbiota caused gradual bone loss in the periodontal tissue (naturally occurring bone loss) but at a much slower rate in that the bone loss instigated by *P. gingivalis* at 6 weeks was comparable in severity to that induced by the commensal microbiota alone over a period of 18 months [24]. When cultivable aerobic and anaerobic commensals were transmitted from SPF to GF mice by co-caging, the GF mice developed bone loss similar to that seen in age-matched SPF mice. This physiological bone loss also depended on complement, since C5aR1-deficient SPF mice had similar periodontal bone levels to those of age-matched normal GF mice, and both of these groups exhibited significantly less naturally occurring bone loss compared to normal SPF mice [24]. In conclusion, complement mediates bone loss, and *P. gingivalis* can alter the complement–host homeostasis dialogue in a way that increases inflammation and bone destruction [3]. Below, the exact molecular mechanisms of complement involvement in dysbiosis and bone loss in the context of *P. gingivalis*–induced periodontitis are discussed.

### P. gingivalis *and immune subversion of complement*

In the subgingival environment, *P. gingivalis* is faced with a survival conundrum: on the one hand, it is imperative to evade immune-mediated killing; on the other hand, *P. gingivalis* needs inflammation to obtain nutrients from inflammatory tissue breakdown. Therefore, the instigation of immune suppression is not a viable option for *P. gingivalis*, even though this tactic is a common evasion strategy of many other pathogens [30]. Below, it is outlined how *P. gingivalis* has ‘resolved’ this paradox and in so doing has benefited the entire microbial community.

A plethora of mechanisms and microbial virulence factors involved in complement evasion has been identified over the years [15,31,32]. With regard to identified mechanisms of complement evasion in periodontitis, the gingipains of *P. gingivalis* are involved in most of them. Gingipains are cysteine proteinases, including lysine-specific gingipain (Kgp) and arginine-specific gingipains (RgpA and RgpB) [33]. These enzymes cleave constituents of periodontal tissue, antibodies, and components of the complement system [34], such as C3 into C3a-like and C3b-like fragments, with extensive further degradation and inactivation [35]. Gingipains also cleave C5 into biologically active C5a and C5a-like fragments much like the host C5 convertase does [35]. Although *P. gingivalis* generates biologically active C5a through direct C5 conversion, the resulting C5b fragment is readily degraded by the gingipains, ostensibly to prevent the formation of the membrane attack complex [35]. It should be noted that inactivation of C3 prevents all three pathways of complement activation [36,37].

A noteworthy feature of gingipains is their dual functionality in targeting and degrading complement proteins [37]. Specifically, the gingipains can exert dose-dependent biphasic effects on complement activation. At low concentrations, the gingipains not only fail to block complement but actually activate the C1 complex and thus trigger the classical pathway [37]. Low concentrations of gingipains are likely to occur at the early stages of *P. gingivalis* colonization. At this stage, the released proteases activate the C1 complex, leading to deposition of C1q on the bacterial surface [37,38]. The ensuing activation of complement may eliminate complement-sensitive commensal bacteria, which could otherwise compete with *P. gingivalis* for niche space and nutrients. At the same time, *P. gingivalis* will not be significantly affected, since it is relatively resistant to complement-mediated opsonization and killing. Specifically, *P. gingivalis* uses one of its gingipains (RgpA) to hijack and attach the circulating C4b-binding protein (a physiological regulator of complement) on its cell surface, thereby acquiring the ability to inhibit the classical and lectin pathways [39]. Moreover, *P. gingivalis* is intrinsically resistant to the lytic action of complement, a property that is attributed to an anionic polysaccharide structure anchored to the cell surface by lipid A (also known as A-LPS) [40,41]. At high concentrations, likely to occur when *P. gingivalis* has established its colonization (hence not needing to be as ‘aggressive’ to its neighbors), the released gingipains can inhibit the bactericidal activity of complement by degrading C3 [37,38]. This function can prevent opsonization of complement-sensitive bacteria in the proximity of *P. gingivalis*, thereby promoting mixed-species biofilm development. Moreover, the diffusion of released gingipains away from the biofilm could generate appropriate enzyme concentrations that could activate complement and hence the flow of inflammatory exudate (gingival crevicular fluid), which, as alluded to above, can provide essential nutrients. In this regard, immunohistochemical studies have indeed detected a concentration gradient of gingipains extending from the subgingival biofilm to the subjacent gingival connective tissue [42].

Local gingipain-induced generation and accumulation of biologically active C5a can activate C5aR1 on leukocytes. *P. gingivalis* also expresses ligands (e.g. lipoproteins) that activate the TLR2–TLR1 complex. Taken together, these features enable *P. gingivalis* to co-activate C5aR1 and TLR2 in both neutrophils and macrophages. In neutrophils, the subsequent crosstalk initiates ubiquitination and proteasomal degradation of the TLR2 adapter MyD88, which prevents a host-protective antimicrobial response [43]. This step requires C5aR1/TLR2-dependent release of transforming growth factor beta 1, which mediates MyD88 ubiquitination *via* the E3 ubiquitin ligase Smurf 1. Moreover, the C5aR1-TLR2 crosstalk activates phosphatidylinositol-3-kinase (Pl3K), which suppresses phagocytosis of *P. gingivalis* and bystander bacteria by inhibiting RhoA GTPase and actin polymerization. At the same time, Pl3K activation upregulates inflammatory cytokine production that is harmless (if not beneficial) to the bacteria. Contrary to MyD88, the alternative TLR2 adapter Mal (MYD88 adapter like) contributes to the above-discussed immune subversion by acting upstream of Pl3K. These experiments were performed in both mouse and human neutrophils and were confirmed *in vivo* in mice [43]. In conclusion, *P. gingivalis* affects neutrophils in ways that promote survival of the microbial community and perpetuation of inflammation.

In macrophages the situation is somewhat different. Here, *P. gingivalis* activates C5aR1 and initiates intracellular Ca^2+^ signaling, which increases synergistically the weak cAMP responses caused by TLR2 activation alone [44]. The activation of cAMP-dependent protein kinase A that follows inhibits nuclear factor κB and glycogen synthase kinase-3β. These inhibitory effects in turn cause suppression of inducible nitric oxide synthase-dependent macrophage killing of *P. gingivalis*. The *P. gingivalis*–induced C5aR1/TLR2 crosstalk in macrophages also inhibits production of interleukin (IL)-12p70 and secondarily interferon gamma (IFN-γ) [45]. However, the induction of other proinflammatory cytokines (such as IL-1β, IL-6, and tumor necrosis factor) by macrophages is enhanced by the *P. gingivalis*–induced C5aR1/TLR2 crosstalk [45]. In summary, *P. gingivalis* inhibits IFN-γ-dependent priming of macrophages and their nitric oxide–dependent pathway for intracellular killing, without affecting the overall ability of macrophages to elicit inflammatory responses.

The selective downregulation of IL-12p70 is also observed when *P. gingivalis* binds complement receptor 3 (CR3) on macrophages [46]. CR3 is a β_2_ integrin (CD11b/CD18) that can bind ligands effectively only when its high-affinity conformation is transactivated, predominantly through inside-out signaling by other host receptors. *P. gingivalis* induces TLR2-mediated transactivation of CR3 through an inside-out pathway that involves Rac1, Pl3K, and cytohesin-1 [47,48]. Activated CR3 reacts with *P. gingivalis* fimbriae and initiates downregulation of IL-12p70 [46]. After binding CR3, *P. gingivalis* not only inhibits IL-12p70 but also enters macrophages in a relatively safe manner [49]. This is probably because CR3 is not linked to potent microbicidal mechanisms, such as those initiated by Fc gamma receptor—mediated phagocytosis [50]. Accordingly, *P. gingivalis* can persist intracellularly in wild-type mouse macrophages much longer than in CR3-deficient macrophages [49].

Intriguingly, although *P. gingivalis* can exploit complement receptors to increase its adaptive fitness in neutrophils and macrophages, it fails to do so in dendritic cells, which appear to use the same receptors to kill this pathogen [51]. The reason is unclear. However, the threat to *P. gingivalis* in the periodontal pocket is first of all neutrophils and macrophages and not dendritic cells, which may be important for instructing T-cell responses to this pathogen [52].

Consistent with its capacity to activate C5aR1 independently of the immunologically activated complement cascade, *P. gingivalis* retained its ability to colonize the periodontium of C3-deficient mice; these mice express normal levels of C5 and C5aR1 required for *P. gingivalis* colonization [53]. Accordingly, *P. gingivalis* could colonize C3-deficient mice. However, its ability to cause dysbiosis in this host was transient, and the microbiota could not be sustained at elevated numbers throughout the experimental period, as observed in similarly treated wild-type control mice [53]. Moreover, *P. gingivalis*–colonized C3-deficient mice had significantly more decreased periodontal inflammation and bone loss than the wild-type controls [53]. These findings suggested that C3 is crucial not only for maximal inflammation and bone loss but also for the long-term persistence of the dysbiotic community, presumably because inflammation − as explained above – is required for nutrient acquisition and the blooming of inflammophilic pathobionts (Figure 2) [7]. Consistent with this notion, the bacterial biomass of subgingival biofilms associated with human periodontitis increases with increasing periodontal inflammation [22], whereas anti-inflammatory treatments in animal models suppress the periodontal bacterial burden [54–56]. The importance of C3 in periodontal disease pathogenesis was definitively confirmed in non-human primates locally treated with a potent C3 inhibitor, the compstatin analog Cp40 (AMY-101) [57]. Indeed, Cp40-treated animals were protected from both *P. gingivalis*/ligature-induced and naturally occurring periodontitis [53,58].

### Is P. gingivalis a keystone pathogen in human periodontitis?

*P. gingivalis* has been termed a keystone pathogen in experimental periodontitis [6,24,59], meaning that this species, at low concentration, has a major influence on the microbial community. In addition to host-response modulation, the keystone pathogenic potential of *P. gingivalis* may also be mediated through its intercellular interactions with other members of the microbial community. Indeed, there is evidence that *P. gingivalis* modulates the commensal oral microbiota through host-independent, direct effects in ways that are consistent with dysbiotic changes [60–62]. Thus, through both host modulation and direct effects on the microbiota, *P. gingivalis* may change its numbers and composition toward a dysbiotic direction and thus accelerate bone destruction. The effect of *P. gingivalis* on the microbial community is greater than should be expected from its low abundance. As mentioned, *P. gingivalis* dramatically affected the oral microbiota when present, even at <0.01% of the total microbiota in the mouse periodontal model.

Although established in the mouse model, the keystone-pathogen concept is consistent with observations in other animal models, some of which are much closer to humans than mice. In rabbits, oral inoculation of *P. gingivalis* induces a shift to a more anaerobic microbiota and an overall increase in the bacterial load of the tooth biofilm [54]. In non-human primates, which naturally harbor *P. gingivalis* in the oral cavity, a gingipain-based vaccine causes a reduction in both the counts of *P. gingivalis* and the total subgingival bacterial load [63], suggesting that the entire biofilm benefits from the presence of *P. gingivalis*. Although this review focuses on *P. gingivalis* and subversion of complement, it should be noted that other periodontitis-associated bacteria, such as *Treponema denticola* and *Tannerella forsythia*, can also effectively evade distinct aspects of the host response [16], suggesting that they can also promote the pathogenicity of the biofilm. Consistent with this notion, oral inoculation of rats with a combination of *P. gingivalis*, *T. denticola*, and *T. forsythia* leads to increased pathogenicity (alveolar bone loss) compared to inoculations with each organism alone [64].

The keystone-pathogen concept for periodontal disease initiation is also consistent with *P. gingivalis* being a quantitatively minor constituent of human periodontitis-associated biofilms. Indeed, contrary to results from early culture-based microbiological studies, most recent metagenomic studies using culture-independent molecular methods show that *P. gingivalis* constitutes a quantitatively minor constituent of human periodontitis-associated biofilms [22,65–67]. However, *P. gingivalis* can also be detected at relatively high abundance in some sites [68]. It is possible that following disease initiation at low *P. gingivalis* colonization levels, the relative abundance of *P. gingivalis* could subsequently increase due to elevated inflammation. This notion requires confirmation in longitudinal studies in human periodontitis patients. However, it is consistent with the reciprocally reinforced interaction between dysbiosis and inflammation that selects for inflammophilic bacteria [7], which in turn is consistent with the ecological plaque hypothesis [69]. A very recent study involving metagenomics sequencing and phylogenetic profiling of the microbial community of human subgingival plaque samples lent support to the keystone pathogen–induced polymicrobial synergy and dysbiosis model [70].

In summary, there is sufficient rationale to suggest that the results from the mouse model could be extrapolated to humans, meaning that *P. gingivalis* may have a keystone-pathogen function in human periodontitis [3]. First, similar to the mouse model, there is a significant increase in the total oral microbial load in humans when periodontal health changes to disease. Second, specific targeting of *P. gingivalis* in both mice and a model (non-human primates) that is very close to humans leads to changes in the entire biofilm. Third, complement function and neutrophil activities (both of which are subverted by *P. gingivalis*) are similar in mice and humans. Fourth, in both human and mouse neutrophils, *P. gingivalis*–instigated C5aR1-TLR2 signaling crosstalk leads to suppression of antimicrobial effects and enhancement of harmless (for the bacteria) inflammation. Fifth, human oral commensals in both health and periodontal disease have the potential to elicit inflammation similar to those in mice. Finally, in both human disease and experimental periodontitis in mice, *P. gingivalis* can be present in low colonization levels compared to the total microbiota. However, the presence of a keystone pathogen can definitively only be determined experimentally through an interventional study. In this regard, if *P. gingivalis* is a keystone pathogen in human periodontitis, then a treatment that can selectively target this pathogen should at the same time affect the entire biofilm and lead to suppression of inflammation and disease development.

Another intriguing question is why the presence of a keystone pathogen, such as *P. gingivalis*, does not always lead to periodontitis. In this regard, *P. gingivalis* may also be detected, albeit less frequently, in the ‘normal’ periodontal microbiota of healthy individuals without causing disease [71–75]. One possible explanation is that there is considerable strain and virulence diversity within the population structure of *P. gingivalis*. Moreover, key *P. gingivalis* virulence factors, including the gingipains that are important for complement subversion, are regulated by local environmental conditions that likely differ among different individuals [3]. It should also be noted that the pathogenicity of *P. gingivalis* can be potentially antagonized by certain members of the microbial community. For instance, *Streptococcus cristatus* inhibits fimbrial gene expression in *P. gingivalis* through the signaling action of arginine deiminase [76]. Consistent with this finding, in human subgingival plaque, the distribution of the two organisms is negatively correlated [77]. Moreover, *S. cristatus* suppresses *P. gingivalis*–induced alveolar bone loss in a mouse model [77]. Therefore, depending on the presence and levels of *S. cristatus* or other antagonistic bacteria, certain individuals may be more resistant to *P. gingivalis*–induced dysbiosis than others are. Another potential explanation is that there might be individuals who can resist the capacity of *P. gingivalis* to convert a symbiotic microbiota into a dysbiotic one by virtue of their intrinsic immune-inflammatory status. For instance, people with alterations in signaling pathways required for immune subversion by *P. gingivalis* should be resistant to the tactics of *P. gingivalis*, thereby counteracting its capacity to precipitate dysbiosis. Therefore, since *P. gingivalis* does not necessarily initiate disease, it should be more accurately considered as a risk factor (as opposed to a causal pathogen) of periodontitis. Moreover, considering that *P. gingivalis* may not be equally pathogenic in all individuals, it could be considered as an opportunistic keystone pathogen.

## Concluding remarks

*P. gingivalis* can affect the commensal microbiota in mice by manipulating complement function. There are good reasons to believe that this may also occur in humans. However, complement targeting by *P. gingivalis* does not exclude that other targets in the immune system can also mediate similar alterations to the oral microbial community. In this respect, *P. gingivalis* likely uses additional mechanisms to protect bystander bacteria and elevate the virulence of the entire microbial community, although most of these other putative mechanisms have not been confirmed *in vivo* in the context of experimental periodontitis. For instance, the ability of *P. gingivalis* to degrade or inactivate antimicrobial peptides *in vitro* could offer protection to bystander bacteria in periodontitis-associated biofilms [78,79]. Moreover, consistent with its ability to modulate actin cytoskeletal rearrangements [43,80], *P. gingivalis* suppresses endocytic events required for *F. nucleatum*–induced NLRP3 inflammasome activation in macrophages [81]. This mechanism may promote the fitness of the microbial community, since inflammasome activation induces pyroptosis (a pro-inflammatory mode of lytic cell death) that protects the host against pathogenic bacteria [82,83]. *P. gingivalis* can additionally manipulate adaptive immune responses by selectively promoting the differentiation and recruitment of CD4+ T-helper 17 cells [84–87], a T-cell subset with a potentially homeostatic role but strongly implicated in periodontal tissue destruction [88,89].

Although complement is unlikely to be the sole target of *P. gingivalis*, there is adequate *in vitro* and *in vivo* mechanistic evidence that complement is subverted by this pathogen in ways that dissociate inflammation (which is enhanced) from immune clearance (which is disarmed). These subversive effects not only protect the microbial community but also generate a nutritionally favorable inflammatory environment [43,90,91]. As periodontitis requires a susceptible host, it should be borne in mind that a keystone pathogen is a risk factor rather than a causal agent. Nevertheless, such pathogens can initiate and/or exacerbate periodontitis in the context of additional risk factors, such as host genotype, stress, diet, or behavior (e.g. smoking) that collectively determine disease susceptibility in an individual. The concepts discussed in this review could be exploited therapeutically for novel and potentially effective approaches to the prevention and treatment of periodontitis. Specifically, a potential strategy could be to target host manipulation strategies of the bacteria (such as the subversion of complement-TLR crosstalk) to restore the host response to a state that can control both inflammation and the periodontal microbiota, thereby promoting periodontal tissue homeostasis. Other implicit strategies could be to interfere with the synergistic microbial mechanisms that drive dysbiosis, such as targeting relevant crucial interspecies interactions [9]. Furthermore, promising new strategies could include those that would favor the selective growth of organisms that are antagonistic to *P. gingivalis*, or other potential keystone pathogens, thereby resisting the transition to a dysbiotic community. Moreover, anti-inflammatory approaches would not only ameliorate destructive inflammation but should also control pathogenic microbial communities by limiting the supply of nutrients through connective tissue breakdown [92,93].
